# From Farms to Markets: Gram-Negative Bacteria Resistant to Third-Generation Cephalosporins in Fruits and Vegetables in a Region of North Africa

**DOI:** 10.3389/fmicb.2017.01569

**Published:** 2017-08-24

**Authors:** Ferielle Mesbah Zekar, Sophie A. Granier, Muriel Marault, Lydia Yaici, Benoit Gassilloud, Charles Manceau, Abdelaziz Touati, Yves Millemann

**Affiliations:** ^1^Laboratoire d'Ecologie Microbienne, Faculté des Sciences de la Nature et de la Vie, Université de Bejaia Bejaia, Algeria; ^2^Laboratory for Food Safety, Agence Nationale de Sécurité Sanitaire de l'Alimentation, de l'Environnement et du Travail (ANSES), Université Paris-Est Maisons-Alfort, France; ^3^Nancy Laboratory for Hydrology, Agence Nationale de Sécurité Sanitaire de l'Alimentation, de l'Environnement et du Travail (ANSES) Nancy, France; ^4^Angers Laboratory for Plant Health, Agence Nationale de Sécurité Sanitaire de l'Alimentation, de l'Environnement et du Travail (ANSES) Angers, France; ^5^Ecole Nationale Vétérinaire d'Alfort, Université Paris-Est Maisons-Alfort, France

**Keywords:** antimicrobial resistance, Gram negative bacteria, third-generation cephalosporin, fruits, vegetables, farm, market, North Africa

## Abstract

The role of food in human exposure to antimicrobial-resistant bacteria is a growing food safety issue. The contribution of fruits and vegetables eaten raw to this exposure is still unclear. The evaluation of contamination levels of fruits, vegetables and the agricultural environment by third-generation cephalosporin (3GC)-resistant Gram-negative bacteria was performed by analyzing 491 samples of fruits and vegetables collected from 5 markets and 7 farms in Bejaia area, north-eastern Mediterranean coast of Algeria. Ninety soil samples and 45 irrigation water samples were also sampled in farms in order to assess them as potential inoculum sources. All samples were investigated at the same time on ceftazidime-containing selective media for 3GC-resistant Gram-negative bacteria detection and on Hektoen media, for *Salmonella* spp. presence. The bacteria isolated (*n* = 30) from fruits and vegetables, soil and irrigation water collected in the farms were almost all non-fermenting bacterial species (*Stenotrophomonas, Acinetobacter, Pseudomonas, Ochrobactrum*) except one strain of *Enterobacter cloacae* and two strains of *Citrobacter murliniae*, isolated on one cucumber and two tomato samples in the same farm. Greater diversity in bacterial species and antimicrobial resistance profiles was observed at markets: *Enterobacteriaceae* (*n* = 41) were as strongly represented as non-fermenting bacteria (*n* = 37). Among *Enterobacteriaceae, E. cloacae* (*n* = 21), and *Klebsiella pneumoniae* (*n* = 13) were the most common isolates. Most of the *K. pneumoniae* isolates were extended-spectrum beta-lactamase (ESBL) producers (*n* = 11). No *Salmonella* spp. was recovered in any sample. This study showed that fruits and vegetables including those which may be eaten up raw constitute a reservoir of 3GC-resistant Gram-negative bacteria and multi-drug resistant-bacteria in general that can be transferred to humans through food. The general public should be informed of this hazard for health in order to encourage good domestic hygiene practices. In addition, further investigation is needed throughout the production chain to enrol professionals in actions to reduce this contamination.

## Introduction

Fresh fruits and vegetables are essential components of a healthy human diet. They provide essential nutrients, such as vitamins, fibers, minerals, and have many health benefits. Therefore, a large number of public health institutions encourage the consumption of fruits and vegetables, and recommend eating at least five fruits and vegetables daily to protect against a range of cardiovascular diseases and cancers (Abadias et al., 2008; Callejón et al., 2015).

Currently, there is a growing demand for these fresh fruits and vegetables for health benefits and at the same time, as lifestyles are changing, current trends show a decrease in the time spent preparing meals (Abadias et al., 2008).

In fact, fresh fruits and vegetables have recently become increasingly recognized as potential vehicles of foodborne diseases (Lynch et al., 2009; Olaimat and Holley, 2012). Many food-borne illness outbreaks in numerous countries have been associated with consumption of contaminated fresh fruits and vegetables, such as fenugreek seed sprouts contaminated with *Escherichia coli* O104:H4 in Europe in 2011, and tomatoes and spinach contaminated with *Salmonella* and *E. coli* O157 in the United States of America in 2013 (AIT, 2013).

The use of antibiotics to treat humans and animals or in agriculture can lead to the selection of antibiotic-resistant bacteria that escape in the environment (Durso and Cook, 2014). Environmental isolates of *Enterobacteriaceae* that have acquired resistance to third-generation cephalosporins (3GCs) constitute a crucial threat for public health as a source of resistance traits for pathogenic bacterial strains that could lead to a failure in antibiotherapy (Blaak et al., 2014).

The role of food in human exposure to antimicrobial-resistant bacteria, including zoonotic pathogens, as well as commensal and environmental bacteria serving as a reservoir for resistance genes, is becoming a growing food safety issue (Campos et al., 2013; Zurfluh et al., 2015). This contamination may thus occur by different means, such as exposure of products to manure, soil, irrigation water, or animal feces harboring these microorganisms. This contamination can also occur during harvesting, post-harvest handling, or distribution due to lack of compliance with elementary food safety and hygiene measures (Abadias et al., 2008; Seo and Matthews, 2014).

*Enterobacteriaceae* are part of the environmental microflora and include common animals' commensals. *Enterobacteriaceae* strains ingested through food may contain Extended Spectrum Beta Lactamases (ESBL) and plasmidic AmpC (pAmpC) genes found on mobile genetic elements. These isolates can then colonize humans or their genes can be transferred to other bacteria during transit in the intestinal tract (Thanner et al., 2016).

There is limited information concerning the nature and ecology of antibiotic-resistant bacteria associated with fresh fruits and vegetables (Ruimy et al., 2010; Blaak et al., 2014; Reuland et al., 2014; Veldman et al., 2014). In addition, no study on that topic has been performed in Algeria to date. The aim of our study was therefore firstly to evaluate the level of contamination of fruits, vegetables and the agricultural environment by 3GC-resistant Gram-negative bacteria; secondly, to search for the source of this contamination; and thirdly, to determine the resistance phenotype of these isolates. To establish whether ESBL-producing *Enterobacteriaceae* on fresh fruits and vegetables reflect those present in the farm environment, fruits and vegetables were collected in farms and at the market as well.

## Materials and methods

### Sampling

A total number of 491 fruit and vegetable samples were collected from seven selected farms and five markets in Bejaia area, Algeria, during a time-period from April 2013 to March 2014.

Farms in the vicinity of Bejaia were contacted in order to ask them access for research purposes. Four large farms and three small family farms allowed full access to their agricultural exploitation. During each visit, samples were taken on any production ready for harvesting. Large farms were each sampled four times; the family farms were sampled only once. A total of 181 samples collected from the farms were analyzed, including 126 tomatoes, 6 zucchini, 12 cucumbers, 21 chilies, and peppers, 1 lettuce, 1 celery, 2 parsleys, 2 mints, 2 garlics, 3 chards, 3 onions, and 2 walnuts. A set of 165 out of these samples originated from large commercial farms while the 16 remaining samples were collected from small family farms (Supplementary Table 1). In addition, 135 samples of soil and irrigation water were sampled in all places (Supplementary Table 1). All farms use poultry droppings, manure, and fertilizer for soil fertilization (Supplementary Table 1).

In parallel, 310 samples were collected in five selected markets located inside the city of Bejaia. During each of the visits to the markets, the sampler behaved like an average Algerian consumer and shopped the most common fruits and vegetables from the regular Algerian regimen. The plant samples included 41 lettuces, 51 tomatoes, 4 cucumbers, 6 celeries, 10 mints, 9 beets, 14 apples, 26 chilies, 28 peppers, 30 grapes, 2 dates, 3 prickly-pears, 11 parsleys, 15 peaches, 24 watermelons, 7 pears, 16 carrots, 7 fennels, 3 turnips, and 3 nectarines (Supplementary Table 2).

For fruit and vegetable samples, we have recorded sample name, date (season), country of origin, number of samples, irrigation water type and the type of fertilization, depending on the availability (Supplementary Tables 1, 2). Information on irrigation water and soil at different farms were also recorded (Supplementary Table 1).

All samples were collected aseptically and then packaged in sterile polyethylene zip bags and transported to the laboratory in aseptic conditions in a cold box within 2 h. All samples were analyzed within 2 h after their arrival at the laboratory. A sharp sterile knife was used to cut samples in sterile trays.

### Microbiological analysis

#### Detection of *Salmonella*

##### Water and soil samples

Isolation of *Salmonella* from irrigation water was carried out by membrane filtration. About 3 liters of irrigation water were filtered through a 0.45 μm filtration membrane. The filter membranes were then placed in 50 mL of buffered peptone water (BPW) (Fluka), and incubated at 37°C for 18 ± 2 h (NF EN; ISO 19250, 2010) for enrichment.

Soil samples (25 g each) were suspended in 225 mL of BPW (Fluka), vigorously shaken and the suspensions were then incubated at 37°C for 18 ± 2 h.

##### Fruit and vegetable samples

*Salmonella* detection was performed following the ISO standard NF EN ISO 6579, 2002. In brief, 25 g of samples of fruit or vegetable were placed aseptically in a sterile plastic bag containing 225 mL of BPW, vigorously shaken and the suspensions were then incubated at 37°C for 18 ± 2 h.

After pre-enrichment, 0.1 mL of each of these incubated samples was then inoculated in 10 mL of Rappaport-Vassiliadis (RV) broth (Fluka), while in parallel 1 mL of the pre-enriched sample was inoculated in 10 mL Muller-Kauffmann (MK) broth (Fluka). Afterwards, these two different broths were incubated at 41.5 ± 1°C and 37°C, respectively for 24 ± 3 h. After incubation, 10 μL of culture from each enrichment broth were streaked on Hektoen enteric agar (HE) plates (Fluka).

Presumptive *Salmonella* isolates were screened biochemically on triple sugar iron (TSI) and identified with an API 20E system (Bio-Merieux, Marcy L'Etoile, France).

#### Isolation and identification of 3GC-resistant gram-negative bacteria

In parallel to *Salmonella* detection, 10 μL of the overnight culture in BPW (see above) was streaked onto MacConkey's agar (MAC) plates (Fluka) supplemented with 8 mg/L of ceftazidime and incubated for 18–24 h at 37°C for isolation of 3GC-resistant Gram-negative bacteria. Vancomycin was added to the medium (8 mg/L) to ensure inhibition of the growth of Gram-positive bacteria. One colony per each morphology and color observed on the agar plate after incubation was preserved. Each preserved isolate, *Enterobacteriaceae* or not, was identified by MALDI-TOF-MS (matrix-assisted laser desorption/ionization time-of-flight mass spectrometry) using a Microflex LT® and Biotype 3.0 software (Bruker Daltonik, GmbH, Germany). Analyses were performed on bacterial cells grown for 21 ± 3 h on plate count agar (PCA) at 36 ± 2°C. Direct spotting of bacteria cells and full protein extraction using absolute ethanol, formic acid and acetonitrile were performed following the manufacturer's recommendations with the aim of obtaining a correct identification score. After drying each spot at room temperature, 1 μL matrix1 HCCA (α-cyano-4-hydroxycinnamic acid in 50% acetonitrile/2.5% trifluoroacetic acid) was added before analysis. A bacterial test standard (BTS, Bruker Daltonik, Germany) was also included bacterial sample lots to assess the efficiency of the process. The identification criteria used were those recommended by the manufacturer. Log scores ≥ 2 were considered reliable for species identifications, log scores ≥ 1.7 and <2.0 were defined as reliable for genus identification, and log scores <1.7 as non-reliable identification.

### Antimicrobial susceptibility testing

Antibiotic susceptibility was tested by the disk diffusion method according to the CLSI protocol (Clinical and Laboratory standards Institute, 2012) on Mueller-Hinton agar (Bio-Rad, Marnes-la-Coquette France). *E. coli* ATCC 25922 was used as a control strain and antimicrobials tested on all isolates were (abbreviations and amounts in parentheses): amoxicillin/clavulanic acid (AMC; 30 μg), ampicillin (AMP; 10 μg), cephalothin (CEF; 30 μg), cefuroxime (CXM; 30 μg), cefamandole (FAM; 30 μg), ceftriaxone (CRO; 30 μg), cefotaxime (CTX; 30 μg), ceftazidime (CAZ; 30 μg), ticarcillin (TIC; 75 μg), cefoxitin (FOX; 30 μg), aztreonam (ATM; 30 μg), cefepime (FEP; 30 μg), temocillin (TMC; 30 μg), ertapenem (ETP; 10 μg), imipenem (IMP; 10 μg), chloramphenicol (CHL, 30 μg), trimethoprim (TMP; 5 μg), sulfonamides (SSS; 300 μg), trimethoprim-sulfamethoxazole (SXT; 1.25+23.75 μg), streptomycin (STR, 10 U), gentamicin (GEN; 10 μg), kanamycin (KAN; 30 UI), tetracycline (TET; 30 UI), tigecycline (TGC; 15 μg), nalidixic acid (NAL; 30 μg), ciprofloxacin (CIP; 5 μg), pefloxacin (PEF, 5 μg), cefotaxime+clavulanic acid (CTC; 30+10 μg), ceftazidime+clavulanic acid (CZC; 30+10 μg; Bio-Rad). Colistin disk (CST; 10 μg) was used on each plate on quality management purposes to ensure the absence of contamination and assess the bacterial identification (Bio-Rad).

Isolates were classified as susceptible, intermediate or resistant according to the clinical interpretative criteria recommended by the CLSI (CLSI, 2013). Multi-drug resistance (MDR) was considered when the isolates were resistant to three or more antibiotic classes (Magiorakos et al., 2012). The detection of ESBL phenotype was performed by either the double disk synergy test (EUCAST, 2013) or combinaison disk test with cefotaxime and ceftazidime (CLSI, 2013; EUCAST, 2013). AmpC phenotype, due to production of an acquired cephalosporinase, was considered present in isolates resistant to cefoxitine and cefotaxime or ceftazidime (EUCAST, 2013). Finally, to be able to detect an ESBL phenotype in presence of a cephalosporinase, combinaison disk test with cefepime were performed and inhibition diameters were compared. For combinaison disk tests, a 5 mm difference at least was considered as positive for ESBL presence (EUCAST, 2013).

### Detection of carbapenemases

To rapidly identify carbapenemase producers in *Enterobacteriaceae*, the Carba NP test and a CIM test (carbapenem inactivation method) were performed according to the methods described by Nordmann et al. (2012) and Van der Zwaluw et al. (2015), respectively.

### Statistical methods

Factors associated with contamination of fruits and vegetables by 3GC-resistant Gram negative bacteria were analyzed using the Chi2 test, according to the place of isolation (farms vs. markets), consumption modes (raw vs. cooked vs. raw/cooked), distance to the soil (above vs. on vs. in the soil) and the season (autumn vs. winter vs. spring vs. summer). Statistical analyses were performed with MS Excel. The Bonferroni correction has also been applied on the set of data: this correction is based on rejecting the null hypothesis if the likelihood of the observed data under the null hypotheses is low. If multiple comparisons are done or multiple hypotheses are tested, the chance of a rare event increases, and therefore, the likelihood of incorrectly rejecting a null hypothesis increases. The Bonferroni correction compensates for that increase by testing each individual hypothesis at a significance level of α/m. where α is the desired overall alpha level and m is the number of hypotheses.

## Results

### Microbiological evaluation of fruits, vegetables, soil, and water samples

In total, 108 different 3GC-resistant Gram-negative bacteria were isolated from all samples (97 isolates from fruits and vegetables and 11 from soil and water); while no *Salmonella* was recovered from any of the samples. A large number of fruits and vegetables, 84 out of 491 (17%; Table 1), were found positive for the occurrence of Gram-negative bacteria resistant to 3GC and other antibiotic classes. In total, 97 different 3GC-resistant Gram-negative bacteria were isolated from 84 positive samples. The contamination frequency of each category of fruits and vegetables varied greatly among selected farms and markets.

**Table 1:** Contamination frequency of fruits and vegetables by 3GC-resistant Gram-negative bacteria.

**Farms/Markets**	**Number plant samples**	**Number of contaminated samples**	**Frequency of contamination**	**95% CI**
Farm no. 1	35	2	6%	
Farm no. 2	41	2	5%	
Farm no. 3	35	0	0%	
Farm no. 4	54	3	6%	
**Total large farms**	**165**	**7**	**4%**	
Farm no. 5	6	4	67%	
Farm no. 6	5	3	60%	
Farm no. 7	5	4	80%	
**Total family farms**	**16**	**11**	**69%**	
**Total on farms**	**181**	**18**	**10%**	**[5.36–14.37%]**^#^
Market Bejaia ville	24	3	13%	
Market Idimco-Bejaia	148	24	16%	
Market Ihadaden-Bejaia	78	23	29%	
Market Lekhmis-Bejaia	48	8	17%	
Market Royal	12	8	67%	
**Total on markets**	**310**	**66**	**21%**	**[16.47–25.53%]**^$^
Grand total	491	84	17%	[13.68–20.32%]

#,$ *Values followed by different signs were found significantly different by Chi2 test (Table S3)*.

In the farms, nearly 10% (18/181) of sampled fruits and vegetables were contaminated with Gram-negative bacteria resistant to 3GC; among them, only 4% (7/165) of the samples collected on the four large commercial farms were found to be positive, while 69% (11/16) of the samples collected from the three small familial farms were contaminated. At the markets, an overall higher and significantly different contamination frequency was found, with 21% (66/310) of the samples found to be positive (Table 1).

The soil and irrigation water contamination frequency (agricultural environment) on farms was also 5% (5/90) and 9% (4/45) respectively.

Fruits and vegetables were found contaminated by a variety of bacteria (Tables 2, 3).

**Table 2:** Description of samples positive for 3GC-resistant Gram-negative bacteria in the Bejaia farms.

**Farms**	**Contaminated plants**	***Enterobacteriaceae***	**Non-fermenting Gram-negative bacteria**
Farm no. 1	Tomato (2)^*^ (fruit)		*Stenotrophomonas maltophilia, Ochrobactrum intermedium*
Farm no. 2	Zucchini (2)^*^ (fruit)		*Acinetobacter* spp., *Pseudomonas* spp.
Farm no. 4	Cucumber (fruit) Tomato (2)^*^ (fruit)	*Citrobacter murliniae Citrobacter murliniae, Enterobacter cloacae*	
Farm no. 5	Lettuce (leaf)		*Acinetobacter* spp.,
			*Pseudomonas* spp.
	Mint (leaf)		*Pseudomonas putida*
	Onion (blub)		*Pseudomonas monteilii*
	Walnuts (fruit)		*Pseudomonas putida*
Farm no. 6	Garlic (corm) Chard (leaf) Parsley (leaf)		*Pseudomonas putida Pseudomonas putida Pseudomonas putida*
Farm no. 7	Garlic (corm)		*Pseudomonas putida*
			*group*
	Onion (blub)		*Acinetobacter pittii*
	Parsley (leaf)		*Pseudomonas* spp.
	Chard (root)		*Pseudomonas monteilii*

* *When indicated, number in parentheses shows number of samples contaminated by one bacterial strain*.

**Table 3:** Description of samples positive for 3GC-resistant Gram-negative bacteria at markets.

**Markets**	**Wilaya of origin**	**Contaminated fruit/vegetable**	**Enterobacteriaceae**	**Non fermentative Gram negative bacteria (Environmental Bacteria)**
Market Bejaia ville	Bejaia	Watermelon (3)^*^ (fruit)	*Enterobacter cloacae, Enterobacter asburiae*	*Comamonas aquatica*
Market Lekhmis	Algiers	Turnip (root)		*Stenotrophomonas maltophilia*
		Fennel (blub and leaf)		*Acinetobacter pittii*
		Carrot (root)		*Acinetobacter pittii*
	Biskra	Celery (leaf)	*Klebsiella pneumoniae*	
		Mint (2)^*^ (leaf)	*Enterobacter aerogenes*	*Acinetobacter pittii*
	Oued Souf	Tomato (fruit)		*Acinetobacter calcoaceticus*
	Sahara	Tomato (fruit)		*Stenotrophomonas maltophilia*
Market Royal	Bejaia	Pepper (fruit)		*Acinetobacter pittii*
		Cucumber (fruit)		*Acinetobacter pittii*
		Carrot (root)		*Acinetobacter pittii*
	Algiers	Beet (root)		*Acinetobacter* spp.
		Lettuce (leaf)		*Acinetobacter pittii*
	Biskra	Parsley (leaf)	*Klebsiella pneumoniae, Enterobacter cloacae, Citrobacter freundii*	*Acinetobacter pittii*
		Tomato (fruit)		*Acinetobacter pittii*
		Celery (leaf)		*Acinetobacter pittii*
Market Idimco	Sétif	Parsley (3)^*^ (leaf)	*Klebsiella pneumoniae*	*Stenotrophomonas maltophilia, Stenotrophomonas maltophilia*
		Mint (2)^*^ (leaf)	*Klebsiella pneumoniae, Klebsiella pneumoniae*	
		Celery (2)^*^ (leaf)		*Stenotrophomonas maltophilia, Ochrobactrum intermedium*
		Lettuce (4)^*^ (leaf)	*Klebsiella pneumoniae*	*Stenotrophomonas maltophilia, Ochrobactrum intermedium, Ochrobactrum intermedium*
	Jijel	Tomato (2)^*^ (fruit)	*Citrobacter murliniae, Klebsiella pneumoniae*	
	Biskra	Tomato (fruit)		*Stenotrophomonas maltophilia*
		Pepper (fruit)		*Stenotrophomonas* spp.
		Chili (fruit)		*Acinetobacter* spp.
	Blida	Peach (fruit)	*Enterobacter cloacae*	
	Tipaza	Tomato (2)^*^ (fruit)	*Klebsiella pneumoniae, Citrobacter freundii*	
	Sahara	Lettuce (leaf)		*Stenotrophomonas maltophilia, Acinetobacter pittii*
	Ain Dafla	Fennel (blub and leaf)	*Citrobacter freundii*	
		Carrot (root)		*Acinetobacter* spp.
	Algiers	Beet (root)		*Stenotrophomonas* spp.
	Skikda	Tomato (fruit)		*Ochrobactrum intermedium*
Market Ihadaden	Tipaza	Tomato (fruit)	*Enterobacter cloacae*	
	Oued Souf	Pepper (fruit)	*Enterobacter cloacae, Kluyvera ascorbata*	*Ochrobactrum intermedium*
	Blida	Nectarine (fruit)	*Enterobacter cloacae*	
	Tiaret	Carrot (root)	*Enterobacter cloacae, Klebsiella pneumoniae*	
	Bejaia	Pear (fruit)	*Enterobacter cloacae*	
		Carrot (2)^*^ (root)	*Enterobacter cloacae, Kluyvera ascorbata*	
		Grape (fruit)	*Enterobacter cloacae*	*Ochrobactrum intermedium*
	Sétif	Beet (2)^*^ (root)	*Enterobacter cloacae, Klebsiella pneumoniae*	*Ochrobactrum intermedium*
		Lettuce (5)^*^ (leaf)	*Enterobacter cloacae, Klebsiella pneumoniae, Enterobacter cloacae, Klebsiella pneumoniae, Enterobacter cloacae*,	*Ochrobactrum intermedium, Stenotrophomonas maltophilia, Ochrobactrum intermedium*
		Chili (fruit)	*Enterobacter cloacae*	*Ochrobactrum intermedium*
	Algiers	Chili (fruit)	*Enterobacter cloacae*	
		Cucumber (fruit)	*Enterobacter cloacae*	
		Apple (fruit)	*Enterobacter cloacae*	
		Peach (2)^*^ (fruit)	*Klebsiella pneumoniae, Enterobacter cloacae*	
		Pear (fruit)	*Enterobacter cloacae*	

* *When indicated, number in parentheses shows number of samples contaminated by one bacterial strain*.

The 108 different bacterial isolates from this study were identified by MALDI-TOF-MS. MALDI-TOF allowed the identification of species for 95 strains, and identification of the genus for 13 other strains with an orientation toward the most likely species.

In the farms, a total number of 19 different bacterial strains were isolated from the 18 positive samples of fruits and vegetables: 16 of them were identified as non-fermenting bacteria, whereas only 3 (3/19) were identified as *Enterobacteriaceae* (Table 2). By contrast, a total number of 78 different bacterial strains were isolated from the 66 positive samples of fruits and vegetables collected at the markets: we found as many samples contaminated with non-fermenting bacteria (37/78) as with *Enterobacteriaceae* (41/78; Table 3).

Only eleven 3GC-resistant bacteria were isolated from samples of soil and water (Table 4). All of them are non-fermenting bacteria.

**Table 4:** Soil and irrigation water samples contaminated with 3GC- resistant Gram-negative bacteria in Bejaia farms.

**Farms**	**Samples**	**Number of samples**	**Number of positive samples**	**Gram-negative bacteria**
Farm no. 1	Soil	18	0	
	Irrigation water	9	1	*Stenotrophomonas maltophilia*
Farm no. 2	Soil	21	1	*Pseudomonas* spp.
	Irrigation water	9	1	*Stenotrophomonas maltophilia*
Farm no. 3	Soil	18	0	
	Irrigation water	9	1	*Acinetobacter pittii*
Farm no.4	Soil	24	0	
	Irrigation water	9	1	*Acinetobacter pittii*
Farm no. 5	Soil	3	2	*Pseudomonas monteilii, Pseudomonas* spp.
	Irrigation water	3	0	
Farm no. 6	Soil	3	1	*Pseudomonas putida, Pseudomonas* spp, *Comamonas aquatica*
	Irrigation water	3	0	
Farm no. 7	Soil	3	1	*Pseudomonas putida_Group*
	Irrigation water	3	0	

In summary, a total of 44 *Enterobacteriaceae* isolates resistant to expanded-spectrum cephalosporins (3GC) were isolated from 491 fruit and vegetable samples (Tables 2, 3), including 21 *E. cloacae*, 13 *K. pneumoniae*, 3 *Citrobacter freundii*, 3 *C. murliniae*, 1 *E. asburiae*, 1 *E. aerogenes*, and 2 *Kluyvera ascorbata*. Among these isolates, only three were collected in the farms: 2 *C. murliniae* isolates (tomato and cucumber) and 1 *E. cloacae* isolate (tomato). All of them were recovered on the same farm (farm no. 4; Table 2). At markets, places of origin of fruits and vegetables contaminated by 3GC-resistant *Enterobacteriaceae* were multiple (Table 3).

Frequency of contamination and species distribution did not differ according to the type of fruits or vegetables. This contamination frequency was different regarding the way the vegetable is to be eaten, i.e., raw or cooked (Table 5). In this case, the contamination of fruits and vegetables usually eaten raw or cooked was significantly different: contamination of vegetables usually eaten cooked was 39%, nevertheless, the contamination of fruits and vegetables usually eaten raw is not negligible, 17%. The fact that fruits or vegetables are in close contact with the soil or not (Table 6) appears to be a determining factor, with fruits and vegetables grown on or in the soil being more contaminated. When considering the season (Table 7), fruits, and vegetables grown in autumn and winter were more contaminated.

**Table 5:** Frequency of fruits and vegetables contaminated with 3GC-resistant Gram-negative bacteria according to usual consumption mode.

**Usual consumption mode**	**Number of samples**	**Number of positive samples**	**Frequency of contamination**	**95% CI**
Raw^a^	158	27	17%	[11.14–22.86%]^$^
Cooked^b^	28	11	39%	[21.1–57.7%]^#^
Raw/Cooked^c^	305	46	15%	[10.99–19.01%]^$^

a *Fruits or vegetables considered usually eaten raw: cucumber, date, prickly pear, lettuce, nectarine, walnut, watermelon, peach, pear, apple, grape*.

b *Fruits or vegetables considered usually eaten cooked: beet, chard, courgette, fennel, turnip*.

c *Fruits or vegetables indifferently eaten raw or cooked: garlic, carrot, celery, mint, onion, parsley, chili, pepper, tomato*.

#,$ *Values followed by different signs were found significantly different by Chi^2^ test (Supplementary Table 3)*.

**Table 6:** Contamination frequency of fruits and vegetables by 3GC-resistant Gram-negative bacteria according to their distance from the soil.

**Contact with soil**	**Number of samples**	**Number of positive samples**	**Frequency of contamination**	**CI 95%**
Above				
• Tree^a^	76	9	12%	$\begin{array}{l} {\left[6.50-21.20\%\right]}^{\#}\\ {\left[4.65-11.35\%\right]}^{\#} \end{array}\}\;{\left[6.02-12.26\%\right]}^{\#}$
• Bush^b^	252	21	8%	
At the soil surface^c^	130	39	30%	[22.12–37.88%]^$^
In the soil^d^	33	15	45%	[29.40–61.60%]^$^

a *Fruits and vegetables considered harvested on trees: date, prickly pear, nectarine, nut, peach, pear, apple, grape*.

b *Fruits and vegetables considered harvested on bushes: chili, pepper, tomato*.

c *Fruits and vegetables considered harvested on the ground: chard, cucumber, lettuce, watermelon, celery, mint, parsley, courgette, fennel*.

d *Fruits and vegetables considered harvested from the soil: beet, garlic, carrot, onion, turnip*.

#,$ *Values followed by different signs were found significantly different by Chi^2^ test (see Supplementary Table 3)*.

**Table 7:** Contamination frequency of fruits and vegetables by 3GC-resistant Gram-negative bacteria depending on the season.

**Season**	**Number of samples**	**Number of positive samples**	**Frequency of contamination**	**CI 95%**
Autumn	78	23	29.49%	[20.10–39.90%]^#^
Winter	113	34	30.08%	[21.62–38.54%]^#^
Spring	52	4	7.69%	[3.00–18.20%]^$^
Summer	248	23	9.27%	[5.44–12.56%]^$^

#,$ *Values followed by different signs were found significantly different by Chi2 test (see Supplementary Table 3)*.

### Antimicrobial susceptibility

A total of 97 suspected 3GC-resistant Gram-negative bacteria isolate from 84 positive fruit and vegetable samples and the 11 non-fermenting bacteria isolates from soil and irrigation water were analyzed using disc diffusion. All isolates were confirmed to be resistant to cefotaxime and ceftazidime.

Resistance profiles were diverse: non-fermenting bacterial strains were resistant to 3rd generation cephalosporins, but with roughly a wild-type profile, while *Enterobacteriaceae* exhibited profiles pointing to acquired resistance to 3rd generation cephalosporins and other antimicrobial classes.

Regarding other beta-lactams, all *Enterobacteriaceae* isolates were resistant to different generations of cephalosporins, including 1st generation cephalosporins (CEF), 2nd Generation cephalosporins [CXM, FAM, except FOX (73%)], and 3rd generation cephalosporins (CTX, CAZ, CRO); 27% of them were resistant to 4th generation cephalosporins (FEP), all of them were resistant to ampicillin and ticarcillin (AMP, TIC), 95% were resistant to AMC, and 78% to monobactam (ATM; Table 8).

**Table 8:** General characteristics of 3GC-resistant *Enterobacteriaceae* obtained from fruits and vegetables in Bejaia, north-eastern Algeria.

**Isolate name**	**Bacterial species**	**Fruits/vegetables**	**Origin**	Antimicrobial resistance profile^*^	**ESBL phenotype**	**AmpC phenotype**
A	*Citrobacter murliniae*	Tomato	Farm no. 4	AMP TIC AMC CEF FAM CXM FOX CTX CAZ CRO	No	Yes
E	*Citrobacter murliniae*	Cucumber	Farm no. 4	AMP TIC AMC CEF FAM CXM FOX CTX CAZ CRO	No	Yes
N5	*Citrobacter murliniae*	Tomato	Market Idimco-Bejaia	AMP TIC AMC CEF FAM CXM FOX CTX CAZ CRO ATM	No	Yes
C5	*Citrobacter freundii*	Tomato	Market Idimco-Bejaia	AMP TIC AMC CEF FAM CXM FOX CTX CAZ CRO ATM	No	Yes
B9	*Citrobacter freundii*	Fennel	Market Idimco-Bejaia	AMP TIC AMC CEF FAM CXM FOX CTX CAZ CRO KAN ERT	No	Yes
K10	*Citrobacter freundii*	Parsley	Market Royal	AMP TIC AMC CEF FAM CXM FOX CTX CAZ CRO ERT	No	Yes
E5	*Klebsiella pneumoniae*	Mint	Market Idimco-Bejaia	AMP TIC AMC CEF FAM CXM CTX CAZ CRO FEP ATM GEN KAN PEF CIP SXT TMP	Yes	No
H5	*Klebsiella pneumoniae*	Parsley	Market Idimco-Bejaia	AMP TIC AMC CEF FAM CXM CTX CAZ CRO FEP ATM GEN KAN PEF CIP SXT TMP	Yes	No
M5	*Klebsiella pneumoniae*	Mint	Market Idimco-Bejaia	AMP TIC AMC CEF FAM CXM CTX CAZ CRO FEP ATM GEN KAN PEF CIP TMP	Yes	No
L5	*Klebsiella pneumoniae*	Tomato	Market Idimco-Bejaia	AMP TIC CEF FAM CXM CTX CAZ CRO FEP ATM GEN PEF	Yes	No
Q5	*Klebsiella pneumoniae*	Lettuce	Market Idimco-Bejaia s	AMP TIC AMC CEF FAM CXM CTX CAZ CRO FEP ATM GEN KAN PEF CIP SXT TMP	Yes	No
K5	*Klebsiella pneumoniae*	Tomato	Market Idimco-Bejaia	AMP TIC AMC CEF FAM CXM CTX CAZ CRO FEP ATM GEN KAN PEF CIP SXT TMP	Yes	No
2D7	*Klebsiella pneumoniae*	Beet	Market Ihadaden-Bejaia	AMP TIC CEF FAM CXM CTX CAZ CRO FEP ATM KAN STR TET TGC PEF SXT TMP SSS CHL	Yes	No
2K7	*Klebsiella pneumoniae*	Peach	Market Ihadaden-Bejaia	AMP TIC CEF FAM CXM CTX CAZ CRO ATM STR PEF SXT TMP SSS CHL	Yes	No
2L7	*Klebsiella pneumoniae*	Carrot	Market Ihadaden-Bejaia	AMP TIC AMC CEF FAM CXM CTX CAZ CRO FEP ATM GEN KAN STR PEF NAL CIP SXT TMP SSS	Yes	No
Z7	*Klebsiella pneumoniae*	Lettuce	Market Ihadaden-Bejaia	AMP TIC AMC CEF FAM FOX CXM CTX CAZ KAN PEF CIP SSS	No	Yes
O7VF	*Klebsiella pneumoniae*	Lettuce	Market Ihadaden-Bejaia	AMP TIC CEF FAM CXM CTX CAZ CRO FEP ATM KAN STR TET TGC PEF SXT TMP SSS CHL	Yes	No
G8	*Klebsiella pneumoniae*	Celery	Market Lekhmis-Bejaia	AMP TIC AMC CEF FAM CXM FOX CTX CAZ CRO ATM STR TET TGC NAL PEF SXT TMP SSS	No	Yes
M10	*Klebsiella pneumoniae*	Parsley	Market Royal	AMP TIC AMC CEF FAM CXM CTX CAZ CRO FEP ATM KAN GEN STR TET SXT TMP SSS	Yes	No
B	*Enterobacter cloacae*	Tomato	Farm no. 4	AMP TIC AMC CEF FAM CXM FOX CTX CAZ CRO ERT PEF	No	Yes
D5	*Enterobacter cloacae*	Peach	Market Idimco-Bejaia	AMP TIC AMC CEF FAM CXM FOX CTX CAZ CRO ATM ERT	No	Yes
17	*Enterobacter asburiae*	Watermelon	Market Bejaia ville	AMP TIC AMC CEF FAM CXM FOX CTX CAZ CRO ATM ERT	No	Yes
14	*Enterobacter cloacae*	Watermelon	Market Bejaia ville	AMP TIC AMC CEF FAM CXM FOX CTX CAZ CRO NAL PEF ERT	No	Yes
A7	*Enterobacter cloacae*	Cucumber	Market Ihadaden-Bejaia	AMP TIC AMC CEF FAM CXM FOX CTX CAZ CRO ATM ERT	No	Yes
J7	*Enterobacter cloacae*	Chili	Market Ihadaden-Bejaia	AMP TIC AMC CEF FAM CXM FOX CTX CAZ CRO ATM ERT	No	Yes
M7	*Enterobacter cloacae*	Carrot	Market Ihadaden-Bejaia	AMP TIC AMC CEF FAM CXM FOX CTX CAZ CRO ATM ERT SXT TMP SSS	No	Yes
N7	*Enterobacter cloacae*	Tomato	Market Ihadaden-Bejaia	AMP TIC AMC CEF FAM CXM FOX CTX CAZ CRO ATM ERT	No	Yes
O7VC	*Enterobacter cloacae*	Lettuce	Market Ihadaden-Bejaia	AMP TIC AMC CEF FAM CXM FOX CTX CAZ CRO ATM ERT	No	Yes
S7	*Enterobacter cloacae*	Pepper	Market Ihadaden-Bejaia	AMP TIC AMC CEF FAM CXM FOX CTX CAZ CRO ATM ERT	No	Yes
W7	*Enterobacter cloacae*	Pear	Market Ihadaden-Bejaia	AMP TIC AMC CEF FAM CXM FOX CTX CAZ CRO ATM ERT	No	Yes
Y7	*Enterobacter cloacae*	Lettuce	Market Ihadaden-Bejaia	AMP TIC AMC CEF FAM CXM FOX CTX CAZ CRO ATM ERT	No	Yes
2B7	*Enterobacter cloacae*	Lettuce	Market Ihadaden-Bejaia	AMP TIC AMC CEF FAM CXM FOX CTX CAZ CRO NAL	No	Yes
X7	*Enterobacter cloacae*	Pear	Market Ihadaden-Bejaia	AMP TIC AMC CEF FAM CXM FOX CTX CAZ CRO ATM ERT	No	Yes
2C7	*Enterobacter cloacae*	Nectarine	Market Ihadaden-Bejaia	AMP TIC AMC CEF FAM CXM FOX CTX CAZ CRO ATM ERT	No	Yes
2J7	*Enterobacter cloacae*	Beet	Market Ihadaden-Bejaia	AMP TIC AMC CEF FAM CXM FOX CTX CAZ CRO ATM ERT NAL PEF	No	Yes
2I7	*Enterobacter cloacae*	Peach	Market Ihadaden-Bejaia	AMP TIC AMC CEF FAM CXM FOX CTX CAZ CRO ATM ERT	No	Yes
2O7	*Enterobacter cloacae*	Carrot	Market Ihadaden-Bejaia	AMP TIC AMC CEF FAM CXM FOX CTX CAZ CRO ATM ERT	No	Yes
2P7	*Enterobacter cloacae*	Grape	Market Ihadaden-Bejaia	AMP TIC AMC CEF FAM CXM FOX CTX CAZ CRO ATM ERT	No	Yes
2Q7	*Enterobacter cloacae*	Chili	Market Ihadaden-Bejaia	AMP TIC AMC CEF FAM CXM FOX CTX CAZ CRO ATM ERT	No	Yes
2R7	*Enterobacter cloacae*	Apple	Market Ihadaden-Bejaia	AMP TIC AMC CEF FAM CXM FOX CTX CAZ CRO ERT	No	Yes
B8	*Enterobacter aerogenes*	Mint	Market Lekhmis-Bejaia	AMP TIC AMC CEF FAM CXM FOX CTX CAZ CRO ATM ERT	No	Yes
L10	*Enterobacter cloacae*	Parsley	Market Royal	AMP TIC AMC CEF FAM CXM FOX CTX CAZ CRO ATM ERT STR TET SXT TMP SSS	No	Yes

* *AMP, ampicillin; TIC, ticarcillin; AMC, amoxicillin-clavulanic acid; CEF, cephalothin; FAM, cefamandole; CXM, cefuroxime; FOX, cefoxitin; CTX, cefotaxime; CAZ, ceftazidime; CRO, ceftriaxone; FEP, cefepime; ATM, aztreonam; ERT, ertapenem; GEN, gentamicin; KAN, kanamycin; STR, streptomycin; TET, tetracycline; TGC, tigecycline; CIP, ciprofloxacin; PEF, pefloxacin; NAL, nalidixic acid; SXT, trimethoprim-sulfamethoxazole; TMP, trimethoprim; SSS, sulfonamides; CHL, chloramphenicol; TEM, temocillin*.

All tested *Enterobacteriaceae* isolates were susceptible to imipenem by disc diffusion. However, *E. cloacae, E. asburiae*, and *E. aerogenes* showed decreased susceptibility to ertapenem (≤23 mm). Nonetheless, these isolates tested negative for carbapenemase production by both Carba NP test and CIM test.

Resistance to non-beta-lactams antibiotics was also encountered: resistance was observed to sulphonamides (29% SXT, 31% TMP, and 20% SSS), aminoglycosides (20% GEN, 27% KAN, and 16% STR), tetracyclines (11% TET, 7% TGC), fluoroquinolones (18% CIP, 36% PEF, and 11% NAL) and phenicols (7% CHL; Table 8).

The proportion of isolates belonging to the different species identified in this study and that are resistant to studied antimicrobial agents are shown in Table 8. ESBL producing bacteria were *K. pneumoniae* strains (*n* = 11), while AmpC producers belonged to various bacterial species (2 *K. pneumoniae*, 3 *C. murliniae* and 3 *C. freundii*).

## Discussion

Our results document the presence of ESBL-producing and AmpC harboring *Enterobacteriaceae* in retail raw fruits and vegetables that were isolated in Algeria, which implies that vegetables may be a source of resistance genes for human microflora. These results are in accordance with other studies identifying vegetables as a possible route for the dissemination of resistance genes in the community (Mesa et al., 2006; Reuland et al., 2014; Ben Said et al., 2015; Zurfluh et al., 2015). Bacteria that were found in this study are not specifically related to fruits and vegetables; they are also frequently isolated from animals or humans. Indeed, *Enterobacteriaceae* such as *E. cloacae, K. pneumonia*, and *Citrobacter* are ubiquitous bacteria that are frequently recovered in the intestines of animals and humans. It is likely that the plants were contaminated indirectly by fecal bacteria from animals during the fertilization process or through direct contact with humans during harvesting, handling and packaging of products due to insufficient hygiene measures (Lynch et al., 2009).

It is interesting to note that no contamination by 3rd generation cephalosporin-resistant *Enterobacteriaceae* was detected in the agricultural environment (water and soil), unlike Ben Said's study (Ben Said et al., 2015), they detected ESBL-producing *Enterobacteriaceae* in the soil, water, as well as on fruits and vegetables. This may be due to the fact that the farms where these bacteria were isolated from use treated wastewater, unlike the farms in our study that use drinking water from wells.

Pignato and al. have reported the potential transfer of antibiotic-resistant bacteria through treated-wastewater (TWW) use in agriculture (Pignato et al., 2009). By contrast, Negreanu et al. did not found so clear relationship and concluded that the impact of TWW-associated bacteria on the soil microbiome is on the whole negligible (Negreanu et al., 2012). In addition, Bartz et al. found that concentration of coliforms in fruits and vegetables matched the contamination of workers' hands rather than water contamination, suggesting that irrigation water is the less relevant sample for detecting the source of contamination of fruits and vegetables (Bartz et al., 2017).

The comparison between the results obtained on fruit and vegetable samples from farms with those from markets might confirm the hypothesis of contamination of fruits and vegetables by humans. In fact, contamination of different fruits and vegetables on different successive vendors' stalls by the same bacterial species was documented. For instance, numerous samples at Ihadaden market were harboring *E. cloacae* (Supplementary Table 4) with the same resistance profile (Table 8).

The comparison between strains isolated from irrigation water and soil with those found on fruits and vegetables leads to conclude that in our study contamination of fruits and vegetables by *Enterobacteriaceae* does not seem to be mainly linked to the agricultural environment. Schwaiger et al. (2011) did not reach the same results and conclusions; they found that contamination of fruits and vegetables is higher at the farm level. They explain this result by the fact that resistance is at the expense of bacterial viability, since vegetables purchased directly at the farm are probably fresher than at the supermarket, and they have not been exposed to stress factors (Schwaiger et al., 2011). This might also be due to different hygiene procedures applied between harvest and sale that are not documented. In addition, we can probably also explain these divergent results by different procedures concerning hygiene either during cultivation or from harvest to the consumer's plate between Algeria and Germany.

The contamination frequency of fruits and vegetables depends on several parameters: type of fruit and/or vegetable, contact with the soil, and season. Here, fruits and vegetables are contaminated with different bacterial species. Fruits and vegetables cultivated and harvested on the surface or in the soil are more commonly contaminated (30 and 45%, respectively), probably due to contact with soil, manure, irrigation water, waste, and animal excrement. This is consistent with findings reported by Ruimy et al. (2010). The variation in fruit and vegetable contamination depending on the season was clearly established. Surprisingly, despite climatic favorable factors in summer and spring contamination was found much higher in winter and autumn (30 and 29%, respectively), than in summer and spring (9 and 8%): the low frequency of contamination of fruits and vegetables in spring could be explained by the fact that fruits and vegetables were harvested in Bejaia and were therefore probably handled to a lesser extent by fewer operators.

This study focused on the detection of 3GC-resistant Gram-negative bacteria from fruits and vegetables, and in addition to 3GC resistance, the isolates showed resistance to between 4 and 10 antimicrobial families used in humans. The detection of human and animal fecal bacterial species with resistance profiles similar to those encountered among hospital isolates is of concern. The abundance of *K. pneumoniae, E. cloacae*, and *Citrobacter* resistant to all β-lactams and other families suggests that fruits and vegetables may constitute a real threat to public health because of the transmissible character of this resistance (Thanner et al., 2016). Even though Ruimy et al. (2010) did not reach the same conclusion, others (Blaak et al., 2014; Reuland et al., 2014; Veldman et al., 2014; Van Hoek et al., 2015) tend to find, like here, various multidrug resistant bacteria in fruits and vegetables. *Stenotrophomonas, Acinetobacter, Pseudomonas, Ochrobactrum*, and *Kluyvera* were frequently encountered. Nevertheless, they are considered to be mostly opportunistic pathogens, associated with patients in poor health conditions, who are frequently immunocompromised.

Many bacteria survive the ingestion process and may contribute to the spread of antimicrobial resistance genes through intestinal tract flora (Schwaiger et al., 2011). The ingestion of bacteria resistant to 3rd generation cephalosporins and their hosting in the intestines can cause the spread of ESBLs and AmpC genes to commensal intestinal flora. Exchanges of these genes between commensal and pathogenic bacteria in the intestinal tract can cause infections that are difficult to treat.

Several studies have shown that consumption of fruits and vegetables can constitute a serious risk for health (Viswanathan and Kaur, 2001; Campos et al., 2013; Warning and Datta, 2013; Blaak et al., 2014; Veldman et al., 2014; Ben Said et al., 2015; Zurfluh et al., 2015). Washing vegetables before eating them raw might reduce not only the risk of infection by pathogenic bacteria, but also the risk of ingesting, hosting and spreading antibiotic-resistant bacteria (Schwaiger et al., 2011).

## Conclusion

In conclusion, poorly washed or insufficiently cooked fruits and vegetables may constitute a public health hazard as they are associated with Gram-negative bacteria resistant to various antibiotics used to treat critical human infections. Fruits and vegetables grown on and in the soil are the most highly contaminated. Contamination frequency of samples from farms is significantly lower than that of samples from markets. This enhanced contamination at the market might reflect poor practices during harvesting and handling of fruits and vegetables.

These results should be communicated to the general public and to fruit and vegetable professionals. First, in order to appreciate the need for good domestic hygiene practices, the consumer has to be aware of the health hazards related to consuming fruits and vegetables that are not properly washed. Second, in order to enroll professionals in actions, close observation and evidence of at-risk practices from harvest to retail are needed.

## Author contributions

FM, SG, AT, and YM: designed the study. FM: Proceeded to sample collection, preparation and bacterial isolation. FM, SG, MM, LY, and BG: Performed laboratory experiments. FM, SG, CM, AT, and YM: Participated to the analyses and discussion of the results.

### Conflict of interest statement

The authors declare that the research was conducted in the absence of any commercial or financial relationships that could be construed as a potential conflict of interest.
