# Supplementary material for: From Farms to Markets: Gram-Negative Bacteria Resistant to Third-Generation Cephalosporins in Fruits and Vegetables in a Region of North Africa
Source: Front Microbiol. 2017 Aug 24;8:1569. doi: 10.3389/fmicb.2017.01569 (PMC5573783; doi:10.3389/fmicb.2017.01569)
Supplement: Supplementary file 1 [file Table1.docx]

Supplementary Material

**From farms to markets: Gram-negative bacteria resistant to third-generation cephalosporins in fruits and vegetables in a region of North Africa**

**Ferielle Mesbah Zekar^1, 2^, Sophie A. Granier^2^, Muriel Marault^2^, Lydia Yaici^1^, Benoit Gassil-loud^3^, Charles Manceau^4^, Abdelaziz Touati^1^ and Yves Millemann^*2, 5^**

*** Corresponding authors: yves.millemann@vet-alfort.fr; +33 1 43 96 71 23**

**Supplementary Table 1. Identified contaminations of fruits and vegetables sampled from Bejaia farms.**

| **Season** | **Farms** | **Samples** | **Number of samples** | **Number of positive samples** | **Isolated species** | **Remarks** |
| --- | --- | --- | --- | --- | --- | --- |
| Spring (26/05/13) | Farm #1  (large commercial farm) | Water | 3 | 1 | *Stenotrophomonas maltophilia* | Potable (Well) |
|  |  | Soil | 6 | 0 | N/A* | Poultry droppings |
|  |  | Tomato | 10 | 0 | N/A |  |
|  | Farm #2  (large commercial farm) | Water | 3 | 1 | *Stenotrophomonas maltophilia* | Potable (Well) |
|  |  | Soil | 3 | 1 | *Pseudomonas spp.* | Poultry droppings |
|  |  |  | 3 | 0 | N/A | Chemical fertilizer |
|  |  |  | 3 | 0 | N/A | Manure |
|  |  | Tomato | 10 | 0 | N/A |  |
|  |  | Zucchini | 6 | 2 | *Acinetobacter spp., Pseudomonas spp.* |  |
|  | Farm #3  (large commercial farm) | Water | 3 | 1 | *Acinetobacter pittii* | Potable (Well) |
|  |  | Soil | 6 | 0 | N/A | Chemical fertilizer |
|  |  | Tomato | 10 | 0 | N/A |  |
|  | Farm #4  (large commercial farm) | Water | 3 | 1 | *Acinetobacter pittii* | Potable (Well) |
|  |  | Soil | 6 | 0 | N/A | Mulching |
|  |  | Tomato | 10 | 1 | *Citrobacter murliniae* |  |
|  |  | Cucumber | 6 | 1 | *Citrobacter murliniae* |  |
| Summer (26/06/13) | Farm #1  (large commercial farm) | Water | 3 | 0 | N/A | Potable (Well) |
|  |  | Soil | 9 | 0 | N/A | Poultry droppings |
|  |  | Tomato | 18 | 2 | *Ochrobactrum intermedium/Stenotrophomonas maltophilia* |  |
|  | Farm #2  (large commercial farm) | Water | 3 | 0 | N/A | Potable (Well) |
|  |  | Soil | 3 | 0 | N/A | Poultry droppings |
|  |  |  | 3 | 0 | N/A | Chemical fertilizer |
|  |  |  | 3 | 0 | N/A | Manure |
|  |  | Tomato | 18 | 0 | N/A |  |
|  | Farm #3  (large commercial farm) | Water | 3 | 0 | N/A | Potable (Well) |
|  |  | Soil | 9 | 0 | N/A | Chemical fertilizer |
|  |  | Tomato | 18 | 0 | N/A |  |
|  | Farm #4  (large commercial farm) | Water | 3 | 0 | N/A | Potable (Well) |
|  |  | Soil | 9 | 0 | N/A | Mulching |
|  |  | Tomato | 18 | 1 | *Enterobacter cloacae* |  |
| Summer (18/07/13) | Farm #1  (large commercial farm) | Water | 3 | 0 | N/A | Potable (Well) |
|  |  | Soil | 3 | 0 | N/A | Poultry droppings |
|  |  | Tomato | 7 | 0 | N/A |  |
|  | Farm #2  (large commercial farm) | Water | 3 | 0 | N/A | Potable (Well) |
|  |  | Soil | 3 | 0 | N/A |  |
|  |  | Pepper | 7 | 0 | N/A |  |
|  | Farm #3  (large commercial farm) | Water | 3 | 0 | N/A | Potable (Well) |
|  |  | Soil | 3 | 0 | N/A |  |
|  |  | Tomato | 7 | 0 | N/A |  |
|  | Farm #4  (large commercial farm) | Water | 3 | 0 | N/A | Potable (Well) |
|  |  | Soil | 9 | 0 | N/A |  |
|  |  | Pepper | 7 | 0 | N/A |  |
|  |  | Chilli | 7 | 0 | N/A |  |
|  |  | Cucumber | 6 | 0 | N/A |  |
| Winter (03/03/14) | Farm #5  (small family farm) | Water | 1 | 0 | N/A | Potable (Well) |
|  |  | Soil | 3 | 2 | *Pseudomonas putida/Pseudomonas monteilii* | Manure |
|  |  | Mint | 1 | 1 | *Pseudomonas putida* |  |
|  |  | Lettuce | 1 | 1 | *Acinetobacter spp., Pseudomonas spp.* |  |
|  |  | Chard | 1 | 0 | N/A |  |
|  |  | Onion | 1 | 1 | *Pseudomonas monteilii* |  |
|  |  | Celery | 1 | 0 | N/A |  |
|  |  | Walnuts | 1 | 1 | *Pseudomonas putida* |  |
|  | Farm #6  (small family farm) | Water | 1 | 0 | N/A | Potable (Well) |
|  |  | Soil | 3 | 1 | *Pseudomonas putida/Pseudomonas monteilii/Comamonas aquatica* | Manure |
|  |  | Walnuts | 1 | 0 | N/A |  |
|  |  | Onion | 1 | 0 | N/A |  |
|  |  | parsley | 1 | 1 | *Pseudomonas putida* |  |
|  |  | Garlic | 1 | 1 | *Pseudomonas putida* |  |
|  |  | Chard | 1 | 1 | *Pseudomonas putida* |  |
| Winter (15/03/14) | Farm #7  (small family farm) | Water | 1 | 0 | N/A | Potable (River) |
|  |  | Soil | 3 | 1 | *Pseudomonas putida_Group* | Manure |
|  |  | Mint | 1 | 0 | N/A |  |
|  |  | Chard | 1 | 1 | *Pseudomonas monteilii* |  |
|  |  | parsley | 1 | 1 | *Pseudomonas spp.* |  |
|  |  | Garlic | 1 | 1 | *Pseudomonas putida_Group* |  |
|  |  | Onion | 1 | 1 | *Acinetobacter pittii* |  |

* N/A not applicable

**Supplementary Table 2. Identified contaminations of fruits and vegetables sampled from Bejaia markets.**

| **Seasons** | **Markets** | **Sellers** | **Origin** | **Samples** | **Number of samples** | **Number of positive samples** | **Isolated species** |
| --- | --- | --- | --- | --- | --- | --- | --- |
| Summer (28/7/13) | Idimco | Seller #1 | Skikda | Tomato | 6 | 0 | N/A* |
|  |  | Seller # 2 | Tipaza | Tomato | 4 | 1 | *Citrobacter murliniae* |
|  |  |  |  | Pepper | 5 | 0 | N/A |
|  |  | Seller #3 | Bejaia | Chili | 4 | 0 | N/A |
|  |  |  | Sétif | Parsley | 3 | 1 | *Stenotrophomonas maltophilia* |
|  |  | Seller #4 | Sétif | Mint | 4 | 1 | *Klebsiella pneumoniae* |
|  |  | Seller #5 | Skikda | Tomato | 6 | 0 | N/A |
|  |  | Seller #6 | Tipaza | Pepper | 6 | 0 | N/A |
|  |  | Seller #7 | Tipaza | Peach | 6 | 0 | N/A |
|  |  |  |  | Tomato | 4 | 1 | *Klebsiella pneumoniae* |
|  |  | Seller #8 | Sétif | Mint | 4 | 1 | *Klebsiella pneumoniae* |
|  |  |  |  | Parsley | 4 | 2 | *Klebsiella pneumoniae/ Stenotrophomonas maltophilia* |
|  |  |  |  | Celery | 4 | 2 | *Stenotrophomonas maltophilia/ Ochrobactrum intermedium* |
|  |  | Seller #9 | Skikda | Tomato | 6 | 1 | *Ochrobactrum intermedium* |
|  |  | Seller #10 | Sétif | Lettuce | 10 | 1 | *Ochrobactrum intermedium* |
|  |  | Seller #11 | Blida | Peach | 6 | 1 | *Enterobacter cloacae* |
|  |  | Seller #12 | Spain | Grapes | 5 | 0 | N/A |
|  |  | Seller #13 | Jijel | Tomato | 4 | 2 | *Klebsiella pneumoniae/Citrobacter freundii* |
|  |  | Seller #14 | Spain | Grapes | 5 | 0 | N/A |
|  |  | Seller #15 | Spain | Grapes | 5 | 0 | N/A |
|  |  | Seller #16 | Sétif | Lettuce | 10 | 3 | *Klebsiella pneumoniae/Ochrobactrum intermedium/Stenotrophomonas maltophilia* |
| Summer (3/8/13) | Centre-ville | Seller #19 | Bejaia | Watermelon | 24 | 3 | *Enterobacter cloacae/Enterobacter asbiriae/Comamonas aquatica* |
| Autumn (28/10/13) | Ihadaden | Seller #20 | Tipaza | Tomato | 3 | 1 | *Enterobacter cloacae* |
|  |  | Seller #21 | Bejaia | Pepper | 3 | 0 | N/A |
|  |  | Seller #22 | Bejaia | Pears | 4 | 1 | *Enterobacter cloacae* |
|  |  | Seller #23 | Bejaia | Carrot | 3 | 2 | *Enterobacter cloacae/Kluyvera ascorbata* |
|  |  | Seller #24 | Oued Souf | Pepper | 4 | 2 | *Enterobacter cloacae/Ochrobactrum intermedium/Kluyvera ascorbata* |
|  |  | Seller #25 | Sétif | Beet | 5 | 2 | *Klebsiella pneumoniae/Enterobacter cloacae/Ochrobactrum intermedium* |
|  |  | Seller #26 | Sétif | Lettuce | 4 | 2 | *Klebsiella pneumoniae/Stenotrophomonas maltophilia/Enterobacter cloacae/Ochrobactrum intermedium* |
|  |  | Seller #27 | Sétif | Lettuce | 4 | 3 | *Klebsiella pneumoniae/Enterobacter cloacae/Ochrobactrum intermedium/Enterobacter cloacae* |
|  |  | Seller #28 | Algiers | Chili | 6 | 1 | *Enterobacter cloacae* |
|  |  | Seller #29 | Algiers | Cucumber | 2 | 1 | *Enterobacter cloacae* |
|  |  | Seller #30 | Sétif | Chili | 4 | 1 | *Enterobacter cloacae/Ochrobactrum intermedium* |
|  |  | Seller #31 | Algiers | Apple | 4 | 1 | *Enterobacter cloacae* |
|  |  | Seller #32 | Algiers | Grapes | 6 | 0 | N/A |
|  |  | Seller #33 | Algiers | Peach | 3 | 2 | *Klebsiella pneumoniae/Enterobacter cloacae* |
|  |  | Seller #34 | Blida | Nectarine | 3 | 1 | *Enterobacter cloacae* |
|  |  | Seller #35 | Bejaia | Tomato | 4 | 0 | N/A |
|  |  | Seller #36 | Bejaia | prickly-pears | 3 | 0 | N/A |
|  |  | Seller #37 | Bejaia | Grapes | 4 | 0 | N/A |
|  |  | Seller #38 | Algiers | Pears | 3 | 1 | *Enterobacter cloacae* |
|  |  | Seller #39 | Bejaia | Grapes | 5 | 1 | *Enterobacter cloacae* |
|  |  | Seller #40 | Tiaret | Carrot | 1 | 1 | *Klebsiella pneumoniae/Enterobacter cloacae* |
| Winter (06/01/14) | Lekhmis | Seller #41 | Sahara | Tomato | 1 | 0 | N/A |
|  |  |  |  | Pepper | 1 | 0 | N/A |
|  |  | Seller #42 | Algiers | Turnip | 1 | 1 | *Stenotrophomonas maltophilia* |
|  |  |  |  | Beet | 1 | 0 | N/A |
|  |  |  |  | Fennel | 1 | 1 | *Acinetobacter pittii* |
|  |  |  | Tipaza | Lettuce | 1 | 0 | N/A |
|  |  |  |  | Apple | 1 | 0 | N/A |
|  |  |  | Media | Carrot | 1 | 0 | N/A |
|  |  |  | Ain Defla | Tomato | 1 | 0 | N/A |
|  |  | Seller #43 | Bejaia | Fennel | 1 | 0 | N/A |
|  |  |  |  | Carrot | 1 | 0 | N/A |
|  |  |  |  | Turnip | 1 | 0 | N/A |
|  |  | Seller #44 | Biskra | Date | 1 | 0 | N/A |
|  |  | Seller #45 | Sahara | Turnip | 1 | 0 | N/A |
|  |  |  | Blida | Apple | 1 | 0 | N/A |
|  |  |  | Bejaia | Lettuce | 1 | 0 | N/A |
|  |  | Seller #46 | Biskra | Pepper | 1 | 0 | N/A |
|  |  |  |  | Chili | 1 | 0 | N/A |
|  |  |  |  | Lettuce | 1 | 0 | N/A |
|  |  |  |  | Tomato | 1 | 0 | N/A |
|  |  | Seller #47 | Biskra | Celery | 1 | 1 | *Klebsiella pneumoniae* |
|  |  |  |  | Mint | 1 | 1 | *Enterobacter aeruginosa* |
|  |  |  |  | Lettuce | 1 | 0 | N/A |
|  |  |  |  | Parsley | 1 | 0 | N/A |
|  |  | Seller #48 | Sahara | Carrot | 1 | 0 | N/A |
|  |  |  |  | Pepper | 1 | 0 | N/A |
|  |  |  |  | Chili | 1 | 0 | N/A |
|  |  | Seller #49 | Oued souf | Tomato | 1 | 1 | *Acinetobacter calcoaceticus* |
|  |  |  |  | Pepper | 1 | 0 | N/A |
|  |  | Seller #50 | Biskra | Mint | 1 | 1 | *Acinetobacter pittii* |
|  |  |  |  | Parsley | 1 | 0 | N/A |
|  |  | Seller #51 | Sahara | Tomato | 1 | 1 | *Stenotrophomonas maltophilia* |
|  |  |  |  | Chili | 1 | 0 | N/A |
|  |  |  |  | Pepper | 1 | 0 | N/A |
|  |  | Seller #52 | Sahara | Lettuce | 1 | 0 | N/A |
|  |  | Seller #53 | Boumerdes | Fennel | 1 | 0 | N/A |
|  |  |  |  | Carrot | 1 | 0 | N/A |
|  |  |  |  | Lettuce | 1 | 0 | N/A |
|  |  | Seller #54 | Algiers | Apple | 1 | 0 | N/A |
|  |  | Seller #55 | Algiers | Carrot | 1 | 1 | *Acinetobacter pittii* |
|  |  |  |  | Lettuce | 1 | 0 | N/A |
|  |  |  |  | Fennel | 1 | 0 | N/A |
|  |  | Seller #56 | Biskra | Chili | 2 | 0 | N/A |
|  |  | Seller #57 | Bejaia | Date | 1 | 0 | N/A |
|  |  | Seller #58 | Bejaia | Parsley | 1 | 0 | N/A |
|  |  | Seller #59 | Bejaia | Apple | 1 | 0 | N/A |
|  |  | Seller #60 | Sétif | Beet | 1 | 0 | N/A |
| Winter (03/02/14) | Idimco | Seller #1 | Sahara | Chili | 1 | 0 | N/A |
|  |  |  |  | Tomato | 1 | 0 | N/A |
|  |  | Seller #2 | Bejaia | Carrot | 1 | 0 | N/A |
|  |  | Seller #3 | Tiaret | Apple | 1 | 0 | N/A |
|  |  | Seller #4 | Media | Apple | 1 | 0 | N/A |
|  |  | Seller #5 | Algiers | Beet | 1 | 1 | *Stenotrophomonas maltophilia* |
|  |  |  | Ain Defla | Carrot | 1 | 1 | *Acinetobacter pittii* |
|  |  |  |  | Fennel | 1 | 1 | *Citrobacter freundii* |
|  |  | Seller #6 | Algiers | Carrot | 1 | 0 | N/A |
|  |  |  | Algiers | Fennel | 1 | 0 | N/A |
|  |  |  | Sahara | Tomato | 1 | 0 | N/A |
|  |  |  | Ain Defla | Lettuce | 1 | 0 | N/A |
|  |  | Seller #7 | Blida | Apple | 1 | 0 | N/A |
|  |  | Seller #8 | Biskra | Tomato | 1 | 0 | N/A |
|  |  |  |  | Pepper | 1 | 0 | N/A |
|  |  |  |  | Chili | 1 | 1 | *Acinetobacter pittii* |
|  |  | Seller #9 | Sahara | Lettuce | 1 | 1 | *Stenotrophomonas maltophilia/Acinetobacter pittii* |
|  |  |  |  | Cucumber | 1 | 0 | N/A |
|  |  | Seller #10 | M'Sila | Carrot | 1 | 0 | N/A |
|  |  | Seller #11 | Biskra | Tomato | 1 | 1 | *Stenotrophomonas maltophilia* |
|  |  | Seller #12 | Biskra | Pepper | 1 | 1 | *Stenotrophomonas maltophilia* |
|  |  |  |  | Chili | 1 | 0 | N/A |
|  |  |  |  | Tomato | 1 | 0 | N/A |
|  |  | Seller #13 | Sahara | Tomato | 1 | 0 | N/A |
|  |  |  |  | Lettuce | 1 | 0 | N/A |
|  |  | Seller #14 | Biskra | Pepper | 1 | 0 | N/A |
|  |  |  |  | Chili | 1 | 0 | N/A |
|  |  |  |  | Lettuce | 1 | 0 | N/A |
|  |  | Seller #15 | Biskra | Chili | 1 | 0 | N/A |
|  |  |  |  | Pepper | 1 | 0 | N/A |
|  |  | Seller #16 | Algiers | Fennel | 1 | 0 | N/A |
|  |  |  |  | Carrot | 1 | 0 | N/A |
|  |  |  |  | Lettuce | 1 | 0 | N/A |
|  |  |  |  | Tomato | 1 | 0 | N/A |
|  |  |  | Oued souf | Carrot | 1 | 0 | N/A |
|  |  | Seller #17 | Biskra | Tomato | 1 | 0 | N/A |
|  |  | Seller #18 | Biskra | Chili | 1 | 0 | N/A |
|  | Royal | Seller #61 | Bejaia | Pepper | 1 | 1 | *Acinetobacter pittii* |
| Winter (11/02/14) | Royal | Seller #61 | Biskra | Chili | 1 | 0 | N/A |
|  |  |  |  | Parsley | 1 | 1 | *Klebsiella pneumoniae/ Enterobacter cloacae/ Citrobacter freundii/Acinetobacter pittii* |
|  |  |  |  | Tomato | 1 | 1 | *Acinetobacter pittii* |
|  |  |  |  | Celery | 1 | 1 | *Acinetobacter pittii* |
|  |  |  | Algiers | Beet | 1 | 1 | *Acinetobacter pittii* |
|  |  |  |  | Lettuce | 1 | 1 | *Acinetobacter pittii* |
|  |  |  |  | Apple | 1 | 0 | N/A |
|  |  |  | Bejaia | Apple | 1 | 0 | N/A |
|  |  |  |  | Cucumber | 1 | 1 | *Acinetobacter pittii* |
|  |  |  |  | Carrot | 1 | 1 | *Acinetobacter pittii* |
|  |  |  | Spain | Apple | 1 | 0 | N/A |

* N/A not applicable

**Supplementary Table 3. Results of conducted statistical analyses.**

|  | **Chi^2^ calculated value** | **P-value (α)** | **Chi² threshold** |
| --- | --- | --- | --- |
| Farms vs Markets | 7.55 | 0.005 (0.05) | 3.84 |
| Raw vs cooked vs raw/cooked | 6.41 | 0.04 (0.017) | 5.99 |
| Raw vs cooked | 4.23 | 0.03 (0.05) | 3.84 |
| Raw vs raw/cooked | 0.22 | 0.63 (0.05) | 3.84 |
| Cooked vs raw/cooked | 6.41 | 0.01 (0.05) | 3.84 |
| Tree vs bush vs on vs in the ground | 31.94 | 5.38 (0.012) | 7.81 |
| Above vs on vs in the ground | 31.50 | 1.44 (0.017) | 5.99 |
| Above vs on the ground | 21.79 | 3.03 (0.05) | 3.84 |
| Above vs in the ground | 22.46 | 2.14 (0.05) | 3.84 |
| Tree vs bush | 0.7 | 0.4 (0.05) | 3.84 |
| Tree vs on the ground | 5.75 | 0.01 (0.05) | 3.84 |
| Tree vs in the ground | 8.84 | 0.002 (0.05) | 3.84 |
| Bush vs on the ground | 21.05 | 4.45 (0.05) | 3.84 |
| Bush vs in the ground | 22.75 | 1.84 (0.05) | 3.84 |
| On vs in the ground | 1.33 | 0.24 (0.05) | 3.84 |
| Autumn vs winter vs spring vs summer | 24.59 | 1.87 (0.012) | 7.81 |
| Autumn vs winter vs spring | 7.15 | 0.02 (0.017) | 5.99 |
| Autumn vs winter vs summer | 20.87 | 2.92 (0.017) | 5.99 |
| Autumn vs spring vs summer | 15.83 | 0.0003 (0.017) | 5.99 |
| Winter vs spring vs summer | 20.07 | 4.36 (0.017) | 5.99 |
| Autumn vs winter | 0.004 | 0.94 (0.05) | 3.84 |
| Autumn vs spring | 6.17 | 0.01 (0.05) | 3.84 |
| Autumn vs summer | 13.85 | 0.0001 (0.05) | 3.84 |
| Winter vs spring | 6.81 | 0.009 (0.05) | 3.84 |
| Winter vs summer | 17.34 | 3.10 (0.05) | 3.84 |
| Spring vs summer | 0.11 | 0.73 (0.05) | 3.84 |

**Supplementary Table 4. List of samples found positive for *Enterobacter cloacae* at Ihadaden market.**

| **Code** | **Seller** | **fruit/vegetable** | **origin** | **seasons** | **species** |
| --- | --- | --- | --- | --- | --- |
| N7 | Seller no. 20 | tomato | Tipaza | autumn | *Enterobacter cloacae* |
| W7 | Seller no. 22 | pear | Bejaia | autumn | *Enterobacter cloacae* |
| S7 | Seller no. 24 | pepper | Oued Souf | autumn | *Enterobacter cloacae* |
| Y7 | Seller no. 26 | lettuce | Sétif | autumn | *Enterobacter cloacae* |
| O7VC | Seller no. 27 | lettuce | Sétif | autumn | *Enterobacter cloacae* |
| 2Q7 | Seller no. 28 | chili | Algiers | autumn | *Enterobacter cloacae* |
| A7 | Seller no. 29 | cucumber | Algiers | autumn | *Enterobacter cloacae* |
| J7 | Seller no. 30 | chili | Sétif | autumn | *Enterobacter cloacae* |
| 2I7 | Seller no. 33 | peach | Algiers | autumn | *Enterobacter cloacae* |
| 2C7 | Seller no. 34 | nectarine | Blida | autumn | *Enterobacter cloacae* |
| X7 | Seller no. 38 | pear | Algiers | autumn | *Enterobacter cloacae* |
| 2P7 | Seller no. 39 | grape | Bejaia | autumn | *Enterobacter cloacae* |
| 2O7 | Seller no. 40 | carrot | Tiaret | autumn | *Enterobacter cloacae* |
